# Enhancer Trajectories in Lineage Commitment: Regulatory Logic of States and Cooperation

**DOI:** 10.3390/biom16010087

**Published:** 2026-01-05

**Authors:** Myunggeun Oh, Seunghwa Jeong, Keunsoo Kang, Seung-Kyoon Kim

**Affiliations:** 1 Department of Convergent Bioscience and Informatics, and Graduate School of Biological Sciences, Chungnam National University, Daejeon 34134, Republic of Korea; 2 Department of Biomedical Sciences, Dankook University, Cheonan 31116, Republic of Korea

**Keywords:** enhancer, chromatin, enhancer state transition, multiple enhancer architecture, lineage commitment, developmental robustness

## Abstract

Cell fate determination depends on precise and timely control of gene expression programs governed by enhancers, which act as central regulatory elements within chromatin landscapes. Recent studies reveal that enhancers occupy distinct functional states, including poised, primed, and active configurations, and that these states dynamically transition during lineage specification. These transitions, in turn, coordinate chromatin accessibility and transcriptional competence, establishing when and how developmental genes become activated. Beyond individual enhancers, some fate-defining loci employ modular and shadow enhancer architectures that cooperatively regulate transcriptional dose, maintain threshold stability, and buffer developmental programs against stochastic and environmental variation. Comparative analyses across neural, cardiac, and hematopoietic systems illustrate how these enhancer modules are selectively deployed to achieve lineage-specific precision and robustness. Furthermore, enhancer timing, persistence, and quantitative thresholds collectively encode developmental tempo and stability, ensuring faithful progression of cell fate transitions. By considering molecular state transitions together with cooperative enhancer architecture, this review organizes current views on how enhancers may help translate transient cues into stable lineage outcomes, thereby linking chromatin dynamics to developmental precision.

## 1. Introduction

Lineage specification requires exquisitely tight spatiotemporal control of gene expression, and this precision fundamentally depends on the coordinated activity of enhancers that modulate transcriptional output in response to developmental cues [1,2,3,4]. Cells must not only activate genes at the right time and place but also establish enhancer competence in advance, maintain alternative programs in reserve, and ensure that enhancer output is directed to the correct promoter with appropriate strength [5]. Viewed from this perspective, enhancer biology provides a conceptual framework for understanding how developmental precision and lineage fidelity are achieved [3,6]. This review focuses on two mechanistic dimensions of enhancer regulation. First, it describes how enhancers transition through distinct functional states, including poised, primed, and active, to confer responsiveness and determine the tempo of lineage activation. Second, it examines how multiple enhancer modules, including modular and shadow architectures, cooperate to set transcriptional dose, maintain threshold stability, and buffer developmental programs against stochastic and environmental variability. Together, these layers of regulation sharpen our understanding of how enhancers can confer both flexibility and robustness during cell fate transitions. Building on this, we systematically examine enhancer state dynamics and cooperative enhancer architecture, assessing the potential and limitations of individual enhancer states and modular logic, and discussing the view that their integration may represent a key axis of enhancer biology and the regulatory processes that mediate lineage commitment.

## 2. What Are Enhancers? Composition & Categories, Identification and Conservation

### 2.1. Enhancer Signatures and Characteristics of Enhancer Subsets

Enhancers are *cis*-regulatory sequences that typically boost promoter activity in a spatiotemporal manner, and they can sometimes act independently of their position and orientation relative to the promoter [7,8,9]. They function by integrating sequence-specific transcription factor (TF) inputs with the local chromatin state and three-dimensional genome architecture, ultimately modulating RNA polymerase II (Pol II) output [8,10]. Enhancer DNA typically harbors clusters of TF motifs that allow cooperative binding, including pioneer TFs that can engage nucleosomal DNA, thereby establishing regulatory competence and scaffolding coactivator assembly [1,4,11]. Upon TF engagement, enhancers recruit chromatin-modifying and remodeling activities and adopt a characteristic chromatin state; histone H3 lysine 4 mono-methylation (H3K4me1) provides a foundational enhancer identity mark, whereas histone H3 lysine 27 acetylation (H3K27ac) distinguishes activation and scales with transcriptional potency. In parallel, other histone tail acetylations, such as histone H3 lysine 9 acetylation (H3K9ac) and histone H3 lysine 14 acetylation (H3K14ac), are frequently enriched at active regulatory elements and similarly reflect the activity of CREB-binding protein/EP300 (CBP/p300) acetyltransferases [2,12,13,14,15,16,17]. These activation-associated chromatin signatures are often coupled to functional outputs at target promoters. For example, active enhancers may contribute to lineage commitment by promoting transcription initiation at target gene promoters, as illustrated by studies of the globin gene loci [18]. Active enhancers are also frequently transcribed into long, often bidirectional enhancer RNAs (eRNAs) [19,20,21]. Although these transcripts were initially debated as to whether they represented mere transcriptional by-products or convenient indicators of enhancer activity, accumulating loss- and gain-of-function studies now support that at least a subset of eRNAs act as bona fide regulatory molecules [22,23,24,25,26,27,28,29]. eRNAs can facilitate promoter-proximal pause release, stabilize enhancer-promoter (E-P) looping, and recruit or allosterically modulate cofactors such as Mediator, cohesin, CBP/p300, bromodomain-containing protein 4 (BRD4), and elongation factors, thereby directly contributing to target gene activation [22,23,24,26,27,28,29]. In this sense, eRNA production is now viewed as one of the mechanistic strategies by which enhancers control the transcriptional output of their cognate genes, rather than simply a passive read-out of enhancer activation.

Before full activation, enhancer regulation can be described along three complementary trajectories, namely poised, primed, and latent, which capture how cells pre-configure, restrain, or newly install regulatory elements during development and in response to environmental cues (Table 1) [5]. As “ready-for-activation” modules, poised and primed enhancers share several core features: they are distal regulatory elements marked by enhancer-associated H3K4me1, exhibit relatively low H3K27ac and minimal eRNA transcription, and are frequently pre-bound by pioneer and lineage-determining transcription factors that maintain chromatin accessibility and transcriptional competence while keeping output restrained until appropriate signals arrive [5,6,15,20]. They diverge, however, in their repressive circuitry and specific chromatin configurations. Poised enhancers, typically located near key developmental regulators, are embedded in Polycomb-controlled domains and are characterized by the combination of H3K4me1 with histone H3 lysine 27 tri-methylation (H3K27me3), often accompanied by histone H2A lysine 119 ubiquitylation (H2AK119ub1) and incorporation of H2A histone family, member Z (H2A.Z), consistent with a Polycomb-restrained yet activation-ready state that can be rapidly converted once lineage cues relieve repression [2,13,15,20,30,31,32]. Primed enhancers, in contrast, are accessible H3K4me1^+^ sites that lack prominent Polycomb-mediated repression and show low levels of H3K27me3; stable occupancy by pioneer and lineage-determining transcription factors predisposes these elements to acquire H3K27ac, increase eRNA synthesis, and transition into high-output active enhancers in a fate- and signal-dependent manner [14,15,20,33,34]. Latent enhancers define a third, mechanistically distinct trajectory: they are initially unmarked and inaccessible, without detectable transcription factor binding or eRNA transcription, but upon stimulation they are engaged de novo, become newly accessible, acquire H3K4me1 together with activation-associated acetylation such as H3K27ac, recruit stimulus-dependent and lineage-specific transcription factors, and initiate eRNA synthesis [35,36,37]. In the following section (Section 3), we discuss how distinct developmental and signal-dependent cues strengthen or attenuate pre-existing active enhancers, resolve “ready-for-activation” enhancer repertoires into lineage-aligned repertoires, and install or preserve latent enhancers as stimulus-responsive modules, thereby dynamically reshaping chromatin landscapes during lineage choice and in the fine-tuning of committed identities.

### 2.2. Methodological Approaches to Enhancer Identification: Strengths and Limitations

Enhancer identification has evolved from low-throughput, candidate-driven reporter assays and conservation-based screens to a broad toolkit spanning chromatin, 3D genome, single-cell, perturbation-based and imaging approaches. Each approach captures a distinct facet of regulatory function and collectively contributes to a more integrated view of gene regulation, yet each remains constrained by characteristic technical and conceptual limitations [10].

Early transgenic and plasmid reporter assays, often guided by sequence conservation and exemplified by resources such as the VISTA Enhancer Browser, provide direct functional readouts in vivo, but they are enriched for a priori selected, frequently highly conserved regions and test sequences outside their native chromatin and three-dimensional context, where integration site and promoter choice can qualitatively influence apparent enhancer activity [38,39]. Complementing these locus-focused assays, chromatin- and transcription-based maps—including DNase-seq, formaldehyde-assisted isolation of regulatory elements sequencing (FAIRE-seq), assay for transposase-accessible chromatin using sequencing (ATAC-seq), chromatin immunoprecipitation sequencing (ChIP-seq), and cleavage under targets and release using nuclease/cleavage under targets and tagmentation (CUT&RUN/CUT&Tag) for p300, Mediator, and histone marks such as H3K4me1 and H3K27ac—have enabled genome-wide nomination of putative enhancers in bulk cell populations and, more recently, in low-input settings [40,41]. Despite their broad utility, these signals may remain largely correlative, be sensitive to antibody quality and peak-calling choices, and miss enhancers that are weakly marked or active only in transient states [10]. Building on bulk chromatin maps, single-cell chromatin accessibility profiling by single-cell ATAC-seq adds a powerful cell-state dimension, resolving lineage- and trajectory-specific enhancer usage and enabling co-accessibility-based E-P linkage, yet the data are extremely sparse and zero-inflated at the single-cell level, typically necessitating cluster-level aggregation that reintroduces population averaging and can render E-P correlations sensitive to technical noise, batch effects, and analytical choices [42,43]. This sparsity reflects the small number of accessible fragments captured per cell, making absence of a signal difficult to interpret as true biological closure. Orthogonal 3D genome mapping technologies, from chromosome conformation capture (3C) and circular chromosome conformation capture (4C) to high-throughput chromosome conformation capture (Hi-C), promoter capture Hi-C, and micrococcal nuclease-based chromosome conformation capture (Micro-C), further refine enhancer maps by assigning distal regulatory elements to promoters based on physical proximity and by revealing higher-order domain architecture, including topologically associating domains (TADs) [44,45,46]. Yet contact frequency may not always provide a perfect proxy for regulatory communication: some statistically robust loops can appear structurally neutral, functionally important E-P pairs may fail to manifest as strong loops at typical resolutions, and population-averaged maps blur heterogeneous and transient interactions while remaining sensitive to normalization artefacts and sequencing-depth limitations [47,48,49]. Complementing these structurally oriented but largely indirect approaches, massively parallel reporter assays (MPRAs), including conventional reporter designs and self-transcribing active regulatory region sequencing (STARR-seq), link sequence and activity for thousands of candidate elements or variants in parallel and have been invaluable for defining motif grammar and non-coding variant effects, but they typically operate in episomal or ectopic genomic contexts that lack native chromatin, insulation, and 3D contacts, so activity measured in the assay can diverge from endogenous behavior and, on its own, provides limited information about target gene identity [10,50]. Pushing closer to causal inference at endogenous loci, clustered regularly interspaced short palindromic repeats (CRISPR)-based perturbation strategies such as CRISPR interference (CRISPRi) and CRISPR activation (CRISPRa) screens, Cas9-mediated deletions, and targeted epigenetic editing now allow systematic testing of enhancer necessity (and to a lesser extent sufficiency) for endogenous gene regulation and phenotypes, particularly when coupled to pooled growth readouts or single-cell transcriptomics, yet interpretation is complicated by guide-dependent off-target binding or cleavage, spreading of repressive or activating chromatin beyond the intended element, the presence of redundant enhancers that can mask regulatory effects, and the relatively coarse perturbation footprint of individual guides [51,52]. Adding a complementary biophysical perspective, live-cell imaging of nascent transcription and labelled genomic loci has illuminated how enhancers shape transcriptional bursting kinetics, but these experiments generally rely on engineered reporters or tagged endogenous loci in a limited set of imaging-amenable models and typically interrogate only one or a few loci per experiment, which constrains generalization [53,54].

Considered together, these strategies provide mutually informative yet largely indirect readouts of enhancer activity, whether from chromatin state, contact probability, reporter output, or genetic perturbation. A more robust framework for enhancer identification will likely emerge from an integrated strategy that combines multimodal single-cell measurements, higher-resolution targeted 3D genome profiling, and carefully controlled CRISPR perturbations, supported by rich molecular and phenotypic readouts. Interpreting these datasets through quantitative, predictive models that explicitly accommodate uncertainty, context, and redundancy will be essential for establishing mechanistic insight.

### 2.3. Evolutionary Dynamics of Enhancer Classes

Functional distinctions among enhancer states are mirrored by clear differences in their evolutionary stability. Latent enhancers, defined by stimulus-dependent establishment, exhibit modest but detectable sequence conservation, suggesting that many reside on conserved DNA scaffolds awaiting inducible activation [35]. Poised enhancers associated with early developmental control genes show stronger conservation of their regulatory architecture, including preserved chromatin and topological features that sustain long-term maintenance at key loci [30,55]. By contrast, active enhancers display low conservation across species and high turnover; comparative H3K27ac maps (e.g., liver across 20 mammals) and phylogenetic modeling indicate that active enhancers are gained or lost roughly twice as fast as promoters, emphasizing lineage-specific activity as the prevailing pattern [56]. When the enhancer is conserved, it tends to occur among pleiotropic elements used across multiple tissues or developmental stages and associated with broadly expressed, loss-of-function (LoF)-intolerant genes [57]. Moreover, co-active sites shared between species often show stronger H3K27ac and chromatin accessibility than species-specific ones, consistent with a more robust regulatory architecture that favors conservation [58]. Overall, genome-scale maps portray active enhancer activity as evolutionarily dynamic, with conservation largely confined to a subset of multi-context regulatory elements.

## 3. Shifting Chromatin Landscapes: Enhancer Dynamics in Lineage Choice

### 3.1. Tuning Active Enhancers at Fate Commitment: Signal-Driven Strengthening and Programmed Attenuation

**Figure 1:**
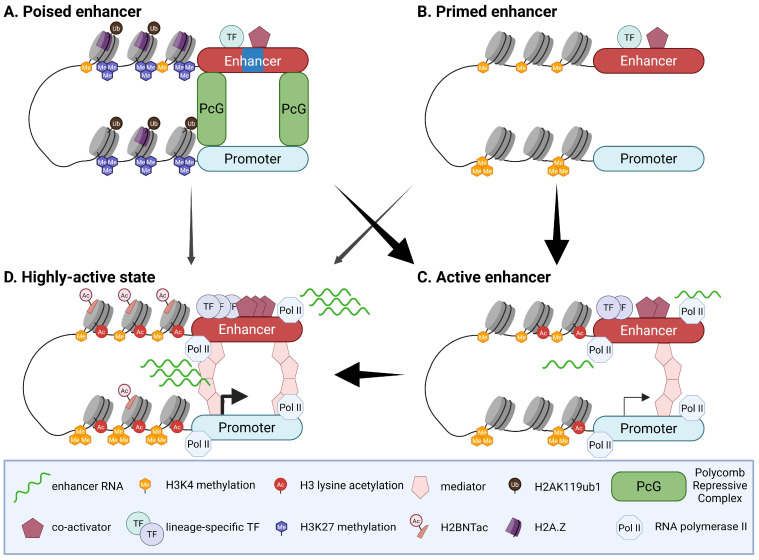
Schematic summary of lineage cue-driven transitions among poised, primed, and active enhancers. Schematic representation illustrating how enhancer states are preset prior to differentiation and remodeled in response to lineage-determining cues. Arrows between panels indicate potential routes of state transition upon developmental signaling, with thicker arrows denoting a higher likelihood of transition under a given cue. (**A**) In the poised state, some E-P wiring is already established within a pre-arranged 3D topology stabilized by Polycomb group (PcG) complexes. Repressive chromatin marks (e.g., H3K27me3, H2AK119ub1), along with the histone variant H2A.Z, are enriched. A lineage-specific TF is bound at the enhancer, but co-activators and Pol II are not yet effectively recruited. (**B**) In the primed state, chromatin becomes more accessible, acquiring H3K4me1 enrichment and permitting partial TF occupancy. Although the lineage-specific TF is present, full co-activator assembly and productive Pol II engagement have not yet occurred. (**C**,**D**) Once lineage-determining cues are delivered, the E-P unit enters an active state. (**C**) Co-activators, Mediator, and Pol II are recruited to the TF-occupied enhancer, histones become acetylated (e.g., H3K27ac, H3K14ac, H3K9ac), and eRNA transcription begins. Mediator-dependent E-P looping stabilizes E-P proximity, resulting in productive transcription of lineage-appropriate genes. (**D**) At the highly active stage, E-P coupling is further strengthened. Acetylation becomes widespread across enhancer and promoter regions (e.g., H2BNTac), additional TFs and Mediator complexes accumulate, eRNA output increases, and Pol II loading at the promoter is elevated, ensuring robust and lineage-stable gene expression.

During fate transitions, commitment cues frequently modulate the output of pre-existing active enhancers on timescales of hours and across successive developmental stages within the same lineage. In these settings, enhancer activity tends not to behave as a simple on/off switch but instead to strengthen or taper in a stage-specific, quantitative manner. In macrophages and estrogen receptor alpha (ERα)-dependent models, stimulus-responsive enhancers show rapid increases in H3K27ac and eRNA levels, and developmental atlases in vertebrate embryos and human erythropoiesis reveal stage-specific enhancer activity that closely parallels changes in acetylation and transcription at the same loci (Figure 1) [36,59,60,61,62]. These observations highlight that commitment cues can rapidly and quantitatively reinforce the strength of pre-existing active enhancers within hours and across successive stages, with changes in histone acetylation and enhancer transcription serving as practical readouts of this graded modulation.

Among the mechanisms underlying such potentiation, CBP/p300-mediated acetylation provides one of the clearest examples of a direct contribution to enhancer strengthening. Lineage- and signal-determining transcription factors (TFs) recruit CBP and p300 to already engaged enhancers, and time-resolved estrogen receptor studies reveal stepwise loading of p300 during enhancer maturation, consistent with signal-dependent catalytic potentiation [59]. Pharmacological inhibition and genetic perturbation further show that the acetyltransferase activity of CBP/p300 is required to maintain H3K27ac at many active enhancers and to sustain associated transcriptional programs: acute inhibition with A-485 triggers rapid loss of H3K27ac and dampens enhancer-dependent transcription, and CBP/p300 and histone deacetylase (HDAC) activities jointly shape H3K27ac dynamics during early development [63,64]. These findings support a causal contribution of CBP/p300 to enhancer strengthening and also suggest that enhancer output may be tuned by chromatin feature-forming modules, particularly via changes in enhancer acetylation (e.g., H3K27ac).

Beyond H3K27ac, histone H2B N-terminal acetylation (H2BNTac) has emerged as a quantitative marker that closely tracks CBP/p300 activity and enhancer strength in vivo [65]. In line with this mechanism, the canonical fate, such as notch homolog protein (Notch), appears to enhance transcription primarily by stimulating the release of paused Pol II at pre-opened regulatory elements, thereby amplifying output from established enhancers rather than creating new accessible regions de novo [66]. Signal-inducible chromatin remodelers can provide an additional layer of control over enhancer competence. Activating protein 1 (AP-1) recruits the switch/sucrose non-fermentable (BAF/SWI-SNF) complex to signal-responsive enhancers, and activity-induced phosphorylation of Brahma-related gene 1 (BRG1) has been shown to increase both basal activity and inducibility at neuronal enhancers [67,68]. Together with live-cell imaging studies, these observations reinforce a model in which stronger enhancers elicit more robust transcriptional bursting. In this framework, enhancer “strength” is expressed primarily through increased burst frequency, rather than larger burst amplitude, during lineage entry [53]. A key unresolved issue, however, is whether comparable shifts in burst dynamics accompany enhancer strengthening in situ, whether achieved through remodeler-dependent increases in competence or through enhanced recruitment of TFs and co-activators.

Feedback between transcription and chromatin provides a further conceptual layer. Acute inhibition of target gene transcription leads to rapid deacetylation of enhancers and a transient collapse of high-output enhancer states, implying that ongoing transcription contributes to the maintenance of histone acetylation and active chromatin [69]. These findings support a self-reinforcing loop in which transcription and acetylation mutually reinforce each other. However, the mechanistic basis of this coupling remains unresolved. It may arise from direct, transcription-linked recruitment of acetyltransferases, from indirect effects driven by changes in TF occupancy and nucleosome turnover, or from more global shifts in nuclear organization. Moreover, the apparent gain of this feedback varies across cell types, loci, and timescales. Further mechanistic dissection will be needed to establish how generally such feedback functions as a principle of enhancer regulation.

By contrast, in lineages that are not ultimately adopted or upon withdrawal of developmental cues, many previously active enhancers undergo stepwise attenuation and, in some cases, decommissioning [70]. Frequently, enhancer acetylation declines as the balance between CBP/p300 and HDAC activity shifts, accompanied by reduced eRNA production [64]. With continued absence of activating cues, lysine-specific demethylase 1 (LSD1) within the nucleosome remodeling and deacetylase (NuRD) complex can remove H3K4me1/2, chromatin accessibility decreases, and the element becomes transcriptionally inactive [70]. Loss of TF occupancy is associated with remethylation of distal regulatory DNA, a change that correlates with more stable long-term silencing and a reduced likelihood of subsequent reactivation [71]. Importantly, these trajectories are heterogeneous: some enhancers remain in low-acetylation, low-output states that retain the potential to be re-engaged, whereas others enter more durable repressive states. This diversity suggests that histone and DNA modifications, when interpreted together with TF network configuration, 3D topology, and signaling history, can act as context-dependent cues that partially foreshadow enhancer competence and long-term fate.

### 3.2. Poised to Choose: Lineage-Dependent Activation at Fate Entry with Poised Topology

In pluripotent contexts, some distal elements near developmental regulators have been reported to exist in a Polycomb-restrained poised state that is already organized in three-dimensional space. Polycomb repressive complex 1 and 2 (PRC1/2) assemble long-range chromatin neighborhoods that position future enhancers and promoters in close proximity before activation, thereby establishing a preconfigured, “ready-on-time” topology that is thought to facilitate rapid transcriptional response [30,72]. When lineage determinants become engaged, poised enhancers often convert swiftly: demethylation of H3K27me3 by lysine-specific demethylase 6A (KDM6A/UTX) or lysine-specific demethylase 6B (KDM6B/JMJD3) is followed by deposition of H3K4me1 and H3K27ac by the coupled histone lysine N-methyltransferase 2D (MLL4/KMT2D)-p300 axis, accompanied by eRNA initiation and relaxation of Polycomb compaction, collectively resulting in the synchronized activation of nearby target genes (Figure 1) [73].

Neural differentiation provides a representative example of this poised-to-active transition. During neural induction, poised enhancers at selected anterior neural loci have been observed to reside within preconfigured Polycomb-dependent chromatin neighborhoods that contact their targets even in pluripotent cells, before transcriptional activation [30]. As cells commit to the neural lineage, some repressive constraints are progressively relieved, accompanied by the emergence of activating features such as Pol II occupancy and H3K27ac. This PRC2-mediated topology enables rapid enhancer activation at the onset of neural fate specification [30]. Mechanistically, JMJD3 recruitment to neural enhancers and neurogenic loci removes H3K27me3, permitting the subsequent action of activating complexes and thereby licensing neural gene expression [74]. In addition, cardiac differentiation provides a complementary example. During early cardiogenesis, UTX/KDM6A is recruited to a subset of poised cardiac enhancers by core cardiac transcription factors such as GATA binding protein 4 (GATA4), NK2 homeobox 5 (NKX2-5), serum response factor (SRF), and T-box transcription factor 5 (TBX5). This process removes H3K27me3 and is essential in vivo for normal heart development [75]. Following demethylation, activation appears to proceed through lineage-coupled histone writers, consistent with the poised-to-active transition described above [73,76]. Genomic context, particularly CpG islands (CGIs), further helps to scaffold both the pre-activation topology and enhancer responsiveness. In poised enhancers, unmethylated CGIs appear to recruit Polycomb complexes and help establish early embryonic chromatin topology at neural developmental loci, thereby priming these regions for timely activation [30,77,78]. Nevertheless, whether such poised-state-mediated pre-topology mechanism can be generalized to a broad spectrum of enhancers or regarded as a universal feature of cellular lineage specification remains unclear and is still a matter of debate; these limitations are discussed in more detail in Section 7.

By comparison, in developmental programs that a cell does not pursue, or when lineage cues are absent or inhibited, poised E-P proximity may either persist as Polycomb-stabilized but nonproductive wiring or become attenuated or reconfigured as fate-specific enhancer hubs emerge. In *Drosophila*, the former scenario, which involves relatively stable physical proximity, is particularly notable. Within Homeobox (*Hox*) clusters, co-repressed loci maintain long-range, Polycomb-dependent colocalization within Polycomb group (PcG) bodies, and these contacts can even strengthen during development despite persistent transcriptional silence [79]. Moreover, embryo-wide chromatin maps in *Drosophila* show that many enhancer loops and local contacts are remarkably stable across tissues [80]. In contrast, during lineage commitment in mammals, contact networks can be more extensively reorganized. For example, when mouse embryonic stem cells (mESCs) enter the neural lineage, the broad Polycomb interaction network constraining developmental loci in pluripotent cells becomes disrupted, and new, lineage-specific enhancer hubs emerge, reflecting loss or rewiring of earlier contacts [81]. In this setting, E-P and E-E interactions appear to be reconfigured in concert with the establishment of lineage-restricted transcriptional programs.

Interestingly, the pre-arranged topologies discussed above may suggest that physical E-P proximity on its own is unlikely to be sufficient to determine transcriptional output, and that changes in contact patterns have limited utility as unambiguous indicators of cause versus consequence in fate decisions. Thus, it remains an open question whether alterations in 3D contacts act as instructive drivers of lineage choice or arise primarily as downstream consequences of changing transcription factor and chromatin states. Accordingly, further work will be required to clarify how contact dynamics are implemented at poised regulatory elements and what functional impact they exert on cell-fate specification and long-term gene regulation.

### 3.3. Fate-Dependent Resolution of Primed Enhancers: Committing or Erasing Predictive Competence

Across developmental lineages, some enhancers are engaged in a biochemically distinct state before robust target gene activation. A recurring observation is that subsets of these “primed” elements later acquire activating features, such as H3K27ac, enhancer transcription, and stable recruitment of lineage-determining transcription factor (LDTF), as cells commit to a particular fate (Figure 1). In this view, primed enhancers may serve as a molecular substrate that can be selectively transitioned to an active state when appropriate combinations of transcription factors, cofactors, and signaling inputs are in place. Pluripotent-to-neural transitions and early embryogenesis illustrate this logic. In ESCs, some distal elements that show a primed-like configuration before lineage choice later gain H3K27ac and other canonical features of enhancer activity upon neural commitment, accompanied by induction of nearby neural genes [14]. Genetic and biochemical studies of MLL3/4 complexes and H3K4 methylation, hallmarks of primed enhancer states, further support a functional role for these writers in enhancer activation and eRNA synthesis across multiple differentiation contexts, although dependence on these marks varies among loci [82,83,84]. Endodermal diversification provides a more mechanistically resolved example. Forkhead box protein A (FOXA) family TFs bind a subset of relatively inaccessible chromatin and “pre-mark” enhancers associated with future pancreatic and hepatic programs, thereby establishing a landscape of lineage-competent elements [84,85,86,87,88]. Upon pancreatic commitment, lineage-determining factors such as PDX1 are recruited to FOXA-primed sites, accompanied by increased H3K27ac and the onset of enhancer transcription [86,87,88]. LoF experiments indicate that FOXA2 is required for efficient priming and subsequent activation of subsets of pancreatic enhancers and for proper lineage progression [87,88], supporting a causal contribution of FOXA-mediated priming to later enhancer engagement. A similar logic is suggested in hematopoiesis. Early multipotent progenitors display broad repertoires of accessible, developmentally relevant enhancers that span multiple potential lineages, and only lineage-matched subsets become strongly engaged as differentiation proceeds, a process often described as “multilineage priming” [89,90,91,92]. The mechanistic resolution of this primed landscape, including how combinatorial LDTF binding and cofactor recruitment restrict which elements become fully active in each fate, is discussed in more detail in Section 6.

When a given lineage is not adopted, primed enhancers follow at least two predominant trajectories. In one, many primed elements remain accessible but exhibit minimal acetylation, persisting as low-output modules that can be rapidly re-engaged if appropriate transcription factor and co-activator combinations arise, as seen for FOXA-primed sites outside the pancreatic lineage and related endodermal intermediates [15,86,93]. In the other, unused enhancer programs appear to be progressively channeled into active silencing or decommissioning pathways that resemble those characterized for pluripotency and other active enhancers [70,71]. Inhibition of LSD1 impairs decommissioning of certain pluripotency enhancers and delays exit from the ESC state [70], indicating that LSD1 contributes to enhancer shutdown, although additional pathways clearly participate.

Taken together, these examples show how primed enhancers can be differentially resolved into activation, persistence in a low-output state, or decommissioning, depending on fate choice and signaling history. Primed chromatin and early TF occupancy thus mark regulatory competence and bias which subsets of enhancers are available for engagement, but they do not by themselves provide reliable certainty about the future activation of any individual element. Section 7 revisits chromatin priming from a quantitative single-cell perspective and outlines mechanistic models for how probabilistic enhancer usage is encoded in primed landscapes.

### 3.4. Latent Enhancers: Stimulus-Responsive Modules in Committed Lineages

Evidence suggests that latent enhancers are typically generated de novo when differentiated cells encounter specific stimuli, rather than being pre-existing elements revealed by activation. In macrophages, stimulation exposes previously unbound and unmarked genomic regions that subsequently acquire enhancer-like features [35]. Upon activation by agents (e.g., Lipopolysaccharide; LPS, Interferon-γ; IFN-γ, Interleukin-4; IL-4), these newly formed regions gain H3K4me1 and H3K27ac, recruit stimulus-activated transcription factors such as nuclear factor kappa-light-chain-enhancer of activated B cells (NF-κB) and AP-1 together with lineage-specific factors, and produce eRNAs, thereby linking chromatin opening to transcriptional activation [36].

Such latent activation mechanisms may exist in specialized lineages and seem particularly prominent in systems whose regulatory logic is highly sensitive to defined signal-coupled transcription factors. In the neural lineage, membrane depolarization of cortical neurons induces thousands of distal sites that gain H3K27ac only after stimulation, consistent with the de novo engagement of activity-dependent enhancers on an otherwise weakly marked chromatin background [94]. In mature pancreatic β cells, the lineage factor PDX1 restrains NF-κB–responsive elements, keeping them relatively inaccessible and hypoacetylated at baseline; reduced PDX1 activity or inflammatory signals relieve this repression, enhance NF-κB binding and chromatin accessibility at latent NF-κB enhancers, and rapidly activate nearby stress-response genes [95]. A similar latent-to-active transition is observed in hormone receptor-positive disease contexts such as ERα-positive breast cancer, where tumor necrosis factor alpha (TNFα) signaling cooperates with NF-κB and FOXA1 to expose latent ERα binding sites that are not used under hormonal stimulation alone but become acetylated and transcriptionally engaged when inflammatory and hormonal pathways converge [96].

Collectively, these findings support a model in which latent enhancers function primarily as stimulus-dependent rapid-response modules layered on top of stable lineage-defining circuitry, rather than as primary drivers of lineage bifurcation or cell-fate choice. Through this dynamic cycle of de novo activation, latent enhancers provide a flexible, signal-responsive regulatory layer that modulates effector gene expression after fate specification and fine-tunes pre-established cellular identities. However, the molecular features that dictate why particular latent enhancers remain selectively closed yet are capable of abrupt pioneer factor-mediated activation upon appropriate stimulation remain incompletely defined, underscoring the need for future studies to elucidate these determinants and integrate them into a more comprehensive framework of signal-dependent enhancer regulation.

## 4. Cooperative Enhancer Architectures Drive and Accelerate Lineage Commitment

### 4.1. Modular Multi-Enhancers Could Set Dose and Buffer the Commitment Transition

**Figure 2:**
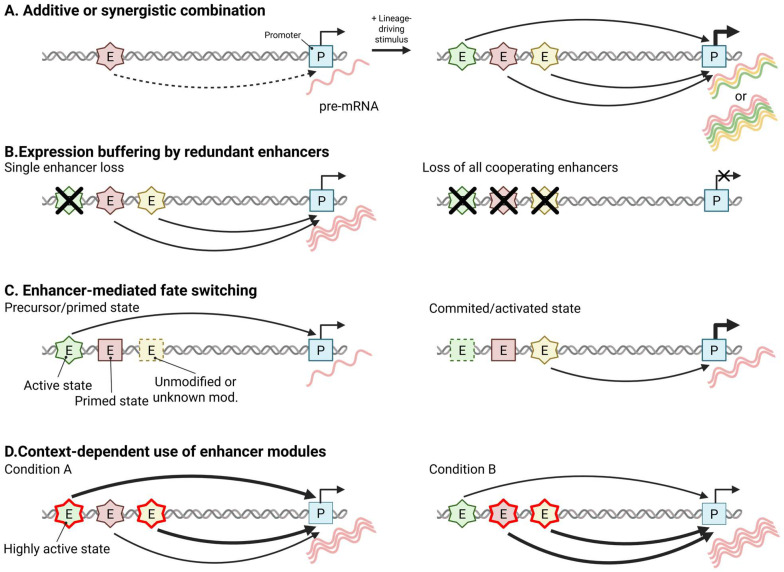
Multiple E-P cooperation and enhancer state dynamics in gene regulation under developmental or conditional cues. Schematic representation illustrating how multiple enhancers linked to a common promoter can act in distinct modes and undergo dynamic state transitions that modulate transcriptional output across differentiation trajectories and in response to physiological or environmental cues. Curved arrows denote transcriptional engagement; arrow thickness indicates interaction strength (bold = strong, intermediate thickness = moderate, dashed = weak). An “X” symbol indicates loss of function of the corresponding enhancer. (**A**) Multiple enhancers can act on the same promoter, and upon appropriate lineage signaling, their combined activity adds to or even exceeds the effect of individual enhancers, thereby amplifying overall transcriptional output. (**B**) Shadow or overlapping enhancers jointly regulate a shared promoter to buffer expression levels; loss of one enhancer produces only a modest reduction because the remaining enhancer can maintain transcriptional activity. (**C**) During lineage progression, pre-existing primed or proto-enhancers (either unmarked or bearing unknown chromatin modifications) are differentially selected to engage a common promoter, thereby fine-tuning gene expression while non-selected enhancers are re-primed for alternative programs or become inactive. (**D**) Under distinct physiological or environmental cues, different enhancer modules within the same locus are preferentially engaged or further strengthened, enabling context-dependent modulation of gene expression.

Multiple enhancers are engaged by developmental and lineage-specific cues. In many characterized loci, these have been observed to (1) help set transcriptional dose through mostly additive, occasionally synergistic, interactions; (2) buffer stochastic and environmental noise and maintain expression thresholds; (3) influence the timing and probability of fate-switch activation; and (4) selectively enhance distinct enhancer modules so that the same locus operates under different subsets in different cellular contexts (Figure 2) [97]. In addition to these quantitative and regulatory roles, multi-enhancer loci often display condition-specific shifts in enhancer state, where distinct modules are established de novo or transition between primed and active configurations depending on developmental cues or signaling inputs (Figure 2C,D). This modular state-switching behavior provides a mechanistic explanation for how different subsets of enhancers can be selectively deployed across developmental or signaling contexts, thereby extending the traditional view that focuses primarily on enhancer dosage and robustness.

Quantitatively, CRISPRi and systematic perturbation analyses reveal that enhancer pairs are predominantly additive, with a smaller fraction exhibiting nonlinear behavior, which is consistent with an approximate arithmetic principle of enhancer cooperation during fate transitions in these experimental systems [97]. Classic in vivo deletions at the β-globin locus, essential for erythropoiesis, confirm dose control through the cumulative contributions of individual 5′ hypersensitive sites within the locus control region (LCR), with compound deletions causing larger, additive-to-synergistic reductions in expression (Figure 2A) [98,99]. Redundancy within enhancer clusters provides an additional layer of robustness, as partial overlap among elements often masks the loss of one enhancer, with phenotypic defects typically appearing only upon double perturbation (Figure 2B) [100]. Threshold enforcement is clearly demonstrated at the Fibroblast growth factor 8 (*Fgf8*) locus in the limb apical ectodermal ridge (AER), where transcriptional control is partitioned across multiple distal enhancers such that deletion of any single element is largely silent, whereas removal of the distal enhancer cluster extinguishes AER *Fgf8* expression and phenocopies limb-specific *Fgf8* ablation, resulting in profound limb developmental defects [101]. At the mechanistic level, enhancer multiplicity interacts with promoter design to modulate transcriptional burst frequency and amplitude, providing a plausible explanation for how multi-enhancer systems might buffer noise during lineage choice in a manner reminiscent of shadow enhancers, without necessarily invoking large-scale chromatin reorganization [53]. Collectively, these studies offer compelling locus-specific evidence that multi-enhancer architectures can regulate transcriptional dosage, thresholds, and noise, yet their generality across genes, tissues, and developmental stages is still uncertain.

Timing and probability of fate commitment are likewise strongly influenced by enhancer activity in several well-studied systems. Work at B-cell lymphoma 11B (*Bcl11b*), runt-related transcription factor 1 (*Runx1*), and interferon regulatory factor 8 (*Irf8*) in T-cell and hematopoietic development shows that some distinct enhancers can mediate slow stochastic *cis*-licensing events, integrate Notch-dependent trans input, and restrict transcription to defined anatomical sites and developmental windows, thereby partitioning specification and maturation phases [102,103,104,105,106]. In these loci, a subset of the available regulatory elements is fully engaged at a given transition, whereas other enhancer modules may remain accessible or weakly marked and can be deployed at later stages or in alternative lineages (Figure 2C) [89,90]. At the network level, lineage-wide profiling of T-cell commitment reveals multi-modular regulatory architectures in which enhancer subsets are preferentially associated with loss of stemness, repression of alternative fates, or stabilization of the chosen lineage, while cross-lineage comparisons and context-dependent studies show that the same gene is wired to distinct enhancer cohorts with internal buffering among convergent elements and that different subsets become more strongly activated in distinct signaling and lineage contexts [107,108].

Single-gene paradigms such as SRY-box transcription factor 10 (*Sox10*), FBJ osteosarcoma oncogene (*fos*), and ETS proto-oncogene 1 (*Ets1*) further illustrate how multi-enhancer architectures distribute regulatory tasks across time, kinds of input, and dosage (Figure 2D) [62,109,110]. In the case of *fos*, for example, depolarization, glutamate receptor activation, or forskolin preferentially potentiates enhancer activation and transcriptional output from a subset of activity-regulated enhancers that are already engaged, whereas other enhancer modules remain active but exhibit comparatively attenuated responses [62]. Across these studies, a modular view is crystallizing in which enhancer ensembles distribute regulatory tasks to tune the timing, anatomical restriction, and robustness of fate decisions at individual loci. In this framework, multi-enhancer loci are not static: distinct modules can be preferentially used under specific developmental or signaling conditions, and can undergo state transitions by switching between primed and active configurations or, in some cases, emerging de novo. Although the prevalence of such architectures remains unclear, and the division of labor and rules of state switching appear to be substantially context dependent, this perspective offers a useful framework for interpreting how multi-enhancer regulation shapes cell fate.

### 4.2. Shadow Enhancers Can Support Robust and Adequate Fate Decisions

Within the broader logic of modular multi-enhancers, shadow enhancers are widely thought to constitute a dedicated robustness layer, comprising two or more distal elements that drive highly overlapping spatiotemporal expression of the same gene to secure dose adequacy and threshold maintenance, a design that is widespread at developmental loci [100,111]. In many cases, rather than partitioning tasks across contexts, they converge on the same output, buffering stochastic and environmental fluctuations and canalizing lineage-defining transcriptional programs through partially distinct TF inputs and kinetics [112,113,114]. In *Drosophila*, quantitative live imaging demonstrates that the majority of primary and shadow enhancer pairs are broadly additive under baseline and moderately perturbed conditions, defining context-dependent rules of enhancer arithmetic that sharpen fate boundaries for patterning genes such as *knirps*, *hunchback*, and *snail* [115]. A subset of shadow pairs also dampens input noise by partially separating upstream fluctuations and maintains low expression noise across a range of temperatures, thereby stabilizing lineage decisions over a substantial window of environmental conditions [114]. Beyond arithmetic and noise control, shadow and duplicated enhancers integrate signals through distinct logic that varies across embryo positions, while individual enhancers modulate transcriptional burst frequency and amplitude to set and refine thresholds essential for cell-identity programs [53,116]. Taken together, these studies establish a *Drosophila* paradigm in which shadow enhancers typically confer additive dose control, noise suppression, and threshold tuning that buffer development across a wide but finite range of perturbations. However, this paradigm is derived from specific loci and experimental regimes and is unlikely to apply uniformly to all regulatory sites or environments: in some contexts, shadow enhancer deletions yield only very minor phenotypic effects, but quantitative imaging has confirmed that sub-additive and interfering modes of enhancer combination also exist, distinct from the additive interactions described above [112,113,115,116]. Therefore, the robustness conferred by shadow enhancers appears at least partially context dependent and limited in magnitude, and under extreme environmental stress or in sensitized genetic backgrounds even multi-enhancer architectures can fail to sustain adequate expression levels and patterning precision (detailed in Section 7) [112,113].

In mammals, similar logic has been proposed to operate in developmental contexts, where some loci carry multiple enhancers with comparable activities that buffer gene expression, so that single enhancer knockouts may produce minimal observable effects, whereas combined losses reveal defects across multiple organs and lineages [100]. Consistent with lineage-defining control, Paired box 6 (*Pax6*) lens induction employs myeloid ecotropic viral integration site (MEIS)-dependent shadow ectoderm enhancer (EE) and SIMO to secure dose and timing, and a human SIMO point mutation disrupts PAX6 autoregulation and causes aniridia [117,118]. Likewise, Sonic hedgehog (*Shh*) expression in oral ectodermal lineages relies on mammal reptile conserved sequence 1 (MRCS1) and mammal fish conserved sequence 4 (MFCS4) redundancy to pattern teeth and tongue papillae, with single deletions having little effect and double knockout revealing buffered control required for epithelial morphogenesis [119]. Finally, the retinal ganglion-cell lineage illustrates threshold maintenance through a promoter-proximal plus remote shadow architecture at atonal BHLH transcription factor 7 (*ATOH7*): deletion of the human remote enhancer causes nonsyndromic congenital retinal non-attachment with optic-nerve aplasia, and the orthologous mouse deletion induces spatiotemporally mis-regulated output [120,121]. Taken together, these findings suggest that, in certain developmental contexts, a shadow enhancer logic operates that integrates dose control, noise attenuation, and robust fate determination in a manner seemingly conserved across species, although it still remains uncertain how pervasive such logic is across multi-enhancer repertoires and diverse developmental settings.

## 5. Convergent and Divergent Enhancer Strategies in Lineage Commitment

### 5.1. Shared Regulatory Logic of Enhancers Across Differentiation Trajectories

Across diverse developmental contexts, enhancer activation during cell-fate transitions seems to emerge as a coordinated interplay between epigenetic pre-marking of regulatory elements and the new establishment of enhancers at previously unmarked loci, with lineage-appropriate transcription factor networks acting as the key arbiters that interpret these pre-marked landscapes and, where necessary, initiate de novo enhancer formation [2,5,85,86,90,93,122,123]. In pluripotent and multipotent progenitors, some future lineage enhancers are already bookmarked by H3K4me1, often in combination with H3K27me3 and increased chromatin accessibility, thereby creating primed or poised configurations that encode developmental competence before overt transcriptional engagement [30,55,86,93]. In parallel, differentiation cues and extracellular stimuli drive the de novo acquisition of enhancer signatures, including H3K4me1, H3K27ac, and binding by LDTFs, at genomic loci that lack canonical enhancer marks in progenitors. This process expands lineage-specific regulatory repertoires in a stage-dependent manner [5,89,122,124,125,126]. Consistent with these observations, comparative chromatin-mapping studies argue that lineage commitment is not adequately explained by either pre-marking or de novo marking alone, but by selective activation and refinement of pre-marked elements together with the stepwise emergence of de novo enhancers, with the relative contribution of each route varying according to tissue, developmental stage and signaling environment [8,127].

At the chromatin level, both poised/primed enhancers and newly formed enhancers converge on a broadly similar activation trajectory dominated by loss of Polycomb-deposited H3K27me3, reinforcement of H3K4me1 and acquisition of H3K27ac, accompanied by enhanced nucleosome turnover and recruitment of co-activators such as p300/CBP [73,76,128]. Polycomb complexes and their demethylases thus operate as a molecular rheostat that keeps developmental enhancers in a repressed yet responsive state, whereas Trithorax/MLL-family methyltransferases and histone acetyltransferases catalyze a rapid and often switch-like transition toward an active enhancer configuration once lineage-inducing cues are received [30,55,83,84,86,93].

Superimposed on these chromatin transitions, lineage-determining and pioneer TFs provide the principal interface between DNA sequence, pre-marked chromatin and de novo enhancer establishment. These factors recognize subsets of primed regulatory elements, stabilize H3K4me1-marked nucleosomes, and recruit histone modifiers and chromatin remodelers, while concurrently opening previously inaccessible chromatin that subsequently acquires enhancer hallmarks and participates in new regulatory hub formation [85,90,122,123]. This dual capacity may allow the same transcription factor networks to interpret pre-existing enhancer landscapes and, where needed, to create new regulatory elements, thereby selecting, amplifying or extinguishing specific enhancer modules in accordance with the lineage trajectory and the prevailing signaling milieu.

### 5.2. Divergent Regulatory Logic of Enhancers Driving Lineage-Specific Differentiation: Focused on Neural, Cardiac, and Hematopoietic Lineages

#### 5.2.1. Neural Lineage: Polycomb-Organized Topology and Pioneer Factor Redeployment

**Figure 3:**
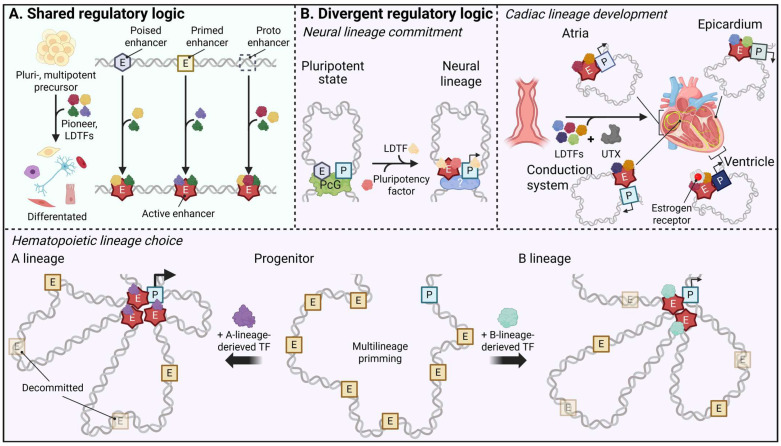
Convergent and divergent enhancer state trajectories and enhancer–promoter hub selection across lineage commitment. Schematic representation illustrating how developing lineages use shared enhancer-state logic together with lineage-distinct mechanisms to assemble distinct E-P hubs and achieve timely and restricted gene expression. (**A**) Convergent mechanism. In pluripotent or multipotent precursors, distal regulatory elements exist in multiple states, including poised and primed enhancers as well as unmarked proto-enhancers; upon lineage induction, various lineage-determining transcription factors (LDTFs) and pioneers engage these elements and drive their transition to active enhancers, thereby organizing lineage-specific regulatory modules. (**B**) Divergent lineage-specific mechanisms. Left panel (neural lineage): Polycomb complexes pre-arrange poised enhancers and their cognate promoters into shared 3D neighborhoods in pluripotent cells, and removal of H3K27me3 together with redeployment of pluripotency factors and neural transcription factors rapidly converts these poised elements into active neurogenic enhancers. However, whether this transition requires the recruitment of additional structural/architectural proteins remains unclear. Right panel (cardiac lineage): In the cardiac lineage, core cardiac transcription factors recruit the H3K27 demethylase UTX to sequence-selected cardiogenic enhancers and selectively license distinct enhancer modules, thereby establishing chamber- and tissue-specific regulatory circuits. Bottom panel (hematopoietic lineage): In the hematopoietic lineage, multipotent progenitors maintain a broad pool of multilineage-primed enhancers from which lineage-restricted transcription factor combinations select and activate appropriate subsets, while non-selected modules are decommissioned, resolving an initially shared primed landscape into distinct enhancer–promoter hubs for each blood lineage.

In pluripotent stem cells, some of the distal regulatory elements located near neural developmental control genes reside in a Polycomb-restrained poised enhancer state marked by H3K4me1 and H3K27me3, in line with the chromatin signatures that define early developmental enhancers in human and mouse embryonic cells [30,55]. In this context, PRC1- and PRC2-mediated chromatin looping positions poised enhancers and their cognate promoters within shared topological neighborhoods already in pluripotent cells, establishing E-P proximity before transcriptional activation of early neural genes (Figure 3) [30,72,81,129]. Upon neural induction, removal of repressive H3K27me3 and the concomitant gain of H3K27ac, often with reinforcement of H3K4me1, convert these elements into active, lineage-specific enhancers, as discussed in Section 3 [14,30,74]. These findings collectively support a model in which Polycomb-organized topology provides a functionally preconfigured scaffold that facilitates rapid transcriptional upshift once repression is lifted, together with additional de novo formation of extensive long-range contacts at the time of enhancer activation [30,81,129]. High-resolution chromatin conformation and imaging studies in pluripotent and early neural cells further demonstrate that many of these Polycomb-anchored hubs persist as relatively stable architectural features during early lineage restriction, even as histone marks, enhancer transcription and gene expression outputs are dynamically remodeled within the same 3D framework [30,55,130].

Recent work adds a second, neural lineage-specific layer by showing that pluripotency factors and early neural transcription factors are repurposed to sculpt this enhancer landscape as cells exit pluripotency: in neural crest and early neural progenitors, pluripotency factors such as SRY-box transcription factor 2 (SOX2) and POU domain, class 5, transcription factor 1 (POU5F1/OCT4) are redeployed to neural enhancers, where they function as pioneer-like factors that cooperate with lineage TFs to open chromatin and stabilize enhancer activity within these pre-existing topological neighborhoods [131,132]. When integrated, these data suggest that in the neural lineage, Polycomb-poised genome architecture and the redeployment of pluripotency-associated pioneer factors cooperate to link 3D organization with transcriptional competence, thereby enabling timely and coordinated activation of neurogenic programs.

#### 5.2.2. Cardiac Lineage: Combinatorial Transcription Factor-UTX Control of Enhancer Activation and Chamber-Specific Wiring

Cardiac specification is organized through an enhancer logic in which lineage-determining transcription factors and the H3K27 demethylase UTX/KDM6A function as an integrated module rather than as independent inputs (Figure 3B). During early cardiogenesis, core cardiac TFs such as GATA4, NKX2-5, TBX5, SRF, and myocyte enhancer factor 2 (MEF2), together with related factors, bind poised cardiogenic enhancers and recruit UTX to these sequence-selected sites. This recruitment erases Polycomb-deposited H3K27me3 and thereby “licenses” the enhancers for activation [75,124]. This TF-directed UTX recruitment emphasizes that TF combinations and local binding grammar determine where demethylase operates, focusing H3K27 demethylation on defined regulatory elements instead of acting in a diffuse, genome-wide manner.

Once repression is relieved, the same TF platforms initiate a coordinated enhancer remodeling cascade [73,76,82,124]. Acting together, cardiac TFs with the activator module both sharpen the activity of pre-primed elements and activate new enhancers at loci that were previously unmarked, so that the cardiomyocyte enhancer repertoire becomes progressively more elaborate as development proceeds. In parallel, enhancer activation is associated with dynamic 3D rewiring: long-range E-P contacts to genes such as muscle myosin 7 (*MYH7*), *NKX2-5*, titin (*TTN*) and sodium voltage-gated channel alpha subunit 5 (*SCN5A*) intensify and spread into multi-enhancer hubs, while H3K27ac/Mediator-rich super-domains accumulate around core cardiogenic loci [49,133,134]. Altogether, TF-UTX-cofactor combinations first define which elements become competent enhancers, and chromatin topology tends to expand as a downstream consequence of this targeted activation.

Within the heart, the same combinatorial logic is redeployed in chamber- and substructure-specific ways (Figure 3B). Atrial and ventricular cardiomyocytes share a common core TF toolkit but deploy it differently across enhancer and regulatory element subsets: TBX5- and COUP transcription factor 2 (COUP-TFII)-biased networks preferentially reinforce atrial-specific genes and their associated regulatory elements, whereas estrogen-related receptors are key activators of ventricular-selective enhancers that contact ventricular-enriched gene programs [135,136,137]. During formation of the cardiac conduction system, enhancers at loci such as *Scn5a*/*Scn10a*, connexin 30.2 (*Cx30.2*) and hyperpolarization-activated cyclic nucleotide gated potassium channel 4 (*Hcn4*) become active in the embryonic atrioventricular canal and emerging conduction tissues, where they are bound by TBX3/TBX5, NKX2-5, GATA4 and other conduction transcription factors, thereby coupling specific TF combinations to the regionalization of conduction gene programs during cardiogenesis [134,138,139,140]. Epicardial and outflow-tract lineages likewise rely on their own enhancer repertoires controlled by TFs such as TBX18, WT1 transcription factor (WT1) and Insulin gene enhancer protein ISL-1 (ISL1), which regulate paracrine signaling, epithelial-to-mesenchymal transition and the formation of coronary vessels and cardiac fibroblasts [141,142,143]. Taken together, observations in the cardiac lineage suggest a dual TF-UTX paradigm in which lineage-specific transcription factor combinations determine where UTX-centered chromatin remodeling is deployed, while chamber- and substructure-specific TF repertoires partition the cardiac genome into multiple, partially overlapping enhancer circuits, emphasizing how chromatin regulated enzyme-mediated remodeling is integrated into combinatorial TF wiring in this setting.

#### 5.2.3. Hematopoietic Lineage: Enhancer Priming, Switching, and Resolution

In the hematopoietic system, multipotent progenitors harbor a broad repertoire of H3K4me1-primed enhancers that anticipate multiple lineage outcomes, as shown by chromatin-state and accessibility maps across early blood development [5,33,89,90,125]. During lineage commitment, specific subsets of these primed elements acquire H3K27ac and increased accessibility in a manner aligned with the adopted fate, whereas enhancers linked to alternative lineages tend to be decommissioned or remain latent (Figure 3B) [33,90,122,125]. A widely studied example is provided by spi-1 proto-oncogene (PU.1): combinations of PU.1 with myeloid partners such as CCAAT/enhancer-binding protein (C/EBP) family members activate myeloid enhancers, whereas PU.1 with B-cell partners such as E2A transcription factor (E2A) or interferon regulatory factors (IRFs) drives B-cell enhancers, all drawn from an initially shared primed enhancer pool in multipotent progenitors [33,89]. These data suggest that enhancer priming in hematopoiesis not only permits but actively encodes alternative lineage trajectories, enabling relatively rapid enhancer selection and reconfiguration in response to cytokine signaling and environmental cues.

Single-cell chromatin accessibility and multiomic analyses reveal that enhancer usage transitions in hematopoiesis tend to be continuous rather than strictly binary, with overlapping enhancer sets marking transient progenitor states at key branch points in the differentiation hierarchy [90,92,122,125]. Dynamic “switching” between primed and active enhancer subsets across these intermediate populations modulates transcriptional dose and the timing of lineage bifurcation, providing finer control than simple on/off regulation [89,91,125]. In erythroid differentiation, for instance, stage-specific activation and subsequent turnover of GATA factor–bound enhancers reshape local 3D chromatin landscapes and align graded increases in transcriptional output with terminal maturation of erythroid gene programs [60,91,144].

Adding to this picture, recent studies integrating enhancer–promoter contact maps with functional genomics suggest that core hematopoietic regulators, including GATA2, RUNX1, and ETS1, are often embedded within multi-enhancer hubs. Within these hubs, quantitative modulation of individual enhancer inputs can fine-tune transcriptional dosage and bias cell-fate decisions, for example, between Th1 and alternative T-cell programs, or between hematopoietic stem cells (HSCs) and downstream progenitor states [102,109,145,146]. Complementary work in mammalian embryos shows that embryonic enhancers controlling hematopoietic regulators can be epigenetically primed before overt blood lineage diversification, linking early enhancer marking to later hematopoietic competence and suggesting that part of the hematopoietic enhancer logic is already established at pre-hematopoietic stages [93]. At the systems level, these coordinated enhancer transitions appear to maintain gene expression thresholds with limited noise, thereby stabilizing lineage boundaries while preserving responsiveness to cytokines, inflammatory signals and stress stimuli in differentiated hematopoietic cells [90,92,147,148,149,150]. In this sense, the hematopoietic lineage has emerged as a prominent model for understanding how enhancer networks integrate temporal, quantitative and architectural control to balance flexibility in progenitors with robustness in differentiated lineages.

Schematic representation illustrating how developing lineages use shared enhancer-state logic together with lineage-distinct mechanisms to assemble distinct E-P hubs and achieve timely and restricted gene expression. (A) Convergent mechanism. In pluripotent or multipotent precursors, distal regulatory elements exist in multiple states, including poised and primed enhancers as well as unmarked proto-enhancers; upon lineage induction, lineage-determining transcription factors (LDTFs) and pioneers engage these elements and drive their transition to active enhancers, thereby organizing lineage-specific regulatory modules. (B) Divergent lineage-specific mechanisms. Left panel (neural lineage): Polycomb complexes pre-arrange poised enhancers and their cognate promoters into shared 3D neighborhoods in pluripotent cells, and removal of H3K27me3 together with redeployment of pluripotency factors and neural transcription factors rapidly converts these poised elements into active neurogenic enhancers. Right panel (cardiac lineage): In the cardiac lineage, core cardiac transcription factors recruit the H3K27 demethylase UTX to sequence-selected cardiogenic enhancers and selectively license distinct enhancer modules, thereby establishing chamber- and tissue-specific regulatory circuits. Bottom panel (hematopoietic lineage): In the hematopoietic lineage, multipotent progenitors maintain a broad pool of multilineage-primed enhancers from which lineage-restricted transcription factor combinations select and activate appropriate subsets, while non-selected modules are decommissioned, resolving an initially shared primed landscape into distinct enhancer–promoter hubs for each blood lineage.

## 6. Functional Implications

### 6.1. Temporal Control and Sequential Activation

During lineage commitment, enhancers are not activated simultaneously but in a temporally ordered fashion that mirrors developmental progression. Early-acting enhancers are often located distally, preloaded with pioneer transcription factors and marked by H3K4me1, while later-acting enhancers acquire the full active enhancer signature, following the activation trajectory outlined in the preceding sections [14,30,73]. This stepwise behavior has been proposed to reflect enhancer hierarchies, in which early-acting modules promote chromatin accessibility and transcriptional competence at some loci, allowing later-acting enhancers to amplify or stabilize target gene expression [146,151].

This temporal layering is therefore thought to contribute to the alignment of enhancer activation with cellular trajectory. In pluripotent stem cells transitioning toward neural or hematopoietic lineages, sequential enhancer engagement correlates with activation of commitment genes only after pluripotency networks have been sufficiently repressed [30,90]. In neural induction, for instance, early distal enhancers preloaded with SRY-box transcription factor 2 (SOX2) or POU domain, class 5, transcription factor 1 (POU5F1) are later joined by lineage-specific TFs such as neurogenic differentiation 1 (NEUROD1) or achaete-scute family bHLH transcription factor 1 (ASCL1), producing a multi-phase activation cascade [131,132,152,153]. At selected loci, the rate and order of enhancer activation have been correlated with differences in the kinetics of promoter engagement and RNA polymerase II loading, consistent with a model in which ordered enhancer use provides a temporal buffer that lowers the probability of premature or ectopic differentiation [154,155]. In this view, enhancer trajectories may encode aspects of developmental tempo and help maintain synchrony between regulatory and transcriptional networks.

### 6.2. Epigenetic Memory and Persistence After Signal Withdrawal

Enhancers can retain molecular memory of previous activation events through residual chromatin marks, bound transcription factors, or partially accessible states. After transient stimulation, a subset of enhancers remains in a “memory-primed” configuration enriched for H3K4me1 but lacking H3K27ac, and these states have been associated with faster and/or stronger reactivation upon secondary cues [35,147]. Such persistent enhancer states are well-documented in macrophages and fibroblasts, where transient inflammatory signals install latent enhancers that reappear upon re-stimulation and, together with other chromatin and metabolic adaptations, constitute a key epigenetic layer of innate immune training [35,36].

Mechanistically, this persistence has been linked to the retention of chromatin accessibility and transcription factor bookmarking at specific genomic loci, a principle that likewise applies to the stabilization of lineage commitment in developmental systems. Once poised enhancers become active, perturbation studies of CBP/p300 and BRD4 demonstrate that these cofactors are required at many enhancers to maintain H3K27ac, sustain transcription, and prevent rapid re-silencing after the initial differentiation cue dissipates [64,156,157,158]. Taken together, these observations are consistent with a model in which persistent enhancer states provide a molecular memory that helps safeguard lineage identity, such that once a cell has transitioned into a particular fate, the enhancer landscape tends to reinforce the newly established transcriptional program despite environmental perturbations or cytokine withdrawal [35,157,159].

### 6.3. Quantitative Thresholds and Robustness of Activation

Enhancers can also function as quantitative integrators that influence whether transcriptional activation surpasses the threshold required for stable lineage specification. Studies using CRISPR interference and single-molecule imaging have shown that transcriptional output scales with enhancer number and strength, with multiple enhancers combining additively or synergistically to reach a critical activation threshold [102,104]. Such scaling relationships have been interpreted as reflecting enhancer density-dependent recruitment of Mediator and coactivators, which may convert analog input signals into switch-like transcriptional responses [160,161]. This architecture enforces binary-like transcriptional outcomes, either maintaining low basal expression or rapidly transitioning into a high-output, lineage-committed state once enhancer input exceeds a defined limit [104].

Shadow enhancer pairs and modular enhancer clusters exemplify this logic, and in many cases, providing redundancy and buffering to preserve transcriptional robustness even under environmental stress or TF dosage variation [107,113,118,119,162]. For example, in *Drosophila* and vertebrate models, loss of a single enhancer within a shadow pair minimally affects output under normal conditions, revealing the importance of enhancer redundancy for developmental precision [113,162]. Conversely, when enhancer activity falls below a critical threshold because of mutations, loss of cooperative modules, or disrupted 3D connectivity, cells can no longer sustain their identity programs; experimental perturbation and human genetic evidence show that such defects lead to developmental arrest or enhanceropathies [163,164,165,166,167,168,169,170,171,172,173].

In this sense, enhancer timing, memory, and activation thresholds are best viewed as forming contributing layers within a tri-layered regulatory logic for developmental control. Timing influences when genes are activated, memory biases how long activation persists, and thresholds help specify the magnitude necessary to maintain fate stability. Together, these parameters convert transient signaling cues into durable, quantitatively balanced transcriptional programs that underlie robust lineage commitment.

## 7. Limitations and Controversies in Models of Developmental Enhancer Regulation

### 7.1. Poised and Primed Chromatin: Limits of a Deterministic Lineage Code

**Figure 4:**
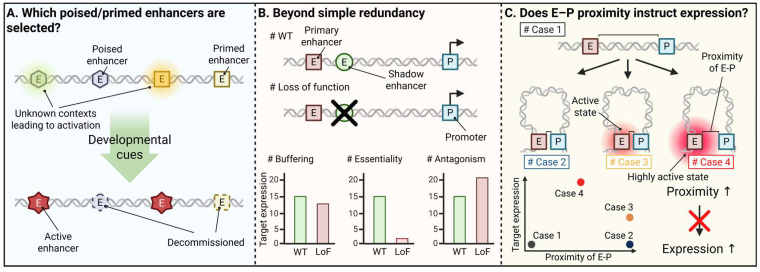
Beyond a deterministic lineage code: how cells select among pre-marked enhancers, organize multi-enhancer logic, and use E-P proximity. Graphical overview of major unresolved questions and conceptual debates in enhancer biology. (**A**) Selection among poised and primed enhancers. At many loci, poised and primed elements create a ready-to-activate regulatory module, yet only a subset is ultimately used during differentiation. The contextual cues and molecular features that determine whether a given element becomes fully activated, remains silent, or is decommissioned are still not well understood. (**B**) Multi-enhancer logic beyond simple redundancy. Primary and shadow enhancers contacting the same promoter can exhibit diverse genetic relationships. Loss-of-function (LoF) of a single enhancer, such as genetic deletion, while the partner enhancer and the rest of the locus remain intact (i.e., an otherwise wild-type background) can reveal not only classical buffering, but also cases where one enhancer is essential for transcription or where the two enhancers act antagonistically. (**C**) E-P proximity: instructive driver or permissive consequence. Enhancer–promoter (E-P) looping is a common architectural feature; however, increased proximity does not necessarily dictate transcription. Instead, E-P topology may often provide an enabling framework, while local regulatory changes (e.g., TF binding and chromatin activation) can drive target-gene expression even when proximity is unchanged or distances increase.

Building on the preceding sections, experimental work in selected systems, most notably in neuronal and hematopoietic lineages, suggests that subsets of poised and primed enhancers can function as preparatory modules that foreshadow future lineage programs and provide a useful, albeit partial, readout of lineage potential (discussed in Section 3 and Section 5). Yet, several genome-wide studies suggest that H3K4me1^+^ (±H3K27me3) configurations, while informative, provide only a partial and context-dependent indication of eventual enhancer deployment within a given lineage and are often better interpreted as a context-dependent correlative marker (Figure 4). Importantly, much of this predictivity is inferred at the population level, and single-cell measurements reveal substantial cell-to-cell variability in whether individual primed elements are actually used [42,89,91,92].

B cell differentiation provides a clear illustration: although hematopoietic stem cells harbor numerous primed enhancers, only ~4% of these elements become active at downstream B cell stages, whereas the majority lose H3K4me1 and close, and some enhancers that are active in mature B cells arise de novo rather than from this primed pool [122]. A similar pattern is observed in hESC-derived endoderm. Along the pancreatic trajectory, many enhancers that pass through a poised state never reach full activation, and numerous poised elements are shared across pancreatic, hepatic, and pulmonary options within the same gut tube population [86]. These observations together suggest that many poised enhancers indicate several possible lineage outcomes rather than predicting one fixed developmental path, which provides a simpler and more flexible interpretation of their function. Consistent with this, single-cell chromatin accessibility studies, including integrated analyses with single-cell transcriptomes, have shown that the regulatory landscape of human hematopoietic differentiation is continuous along trajectories and heterogeneous within immunophenotypically similar progenitors, with accessibility at putative regulatory sites varying across individual cells [42,92].

Together with bulk enhancer-mapping studies showing that primed and active enhancers are used in a dynamic, stage-specific manner during hematopoiesis [91], these observations support the idea that primed regulatory landscapes are resolved during lineage commitment in a probabilistic, rather than deterministic, way. Moreover, primed states can reflect developmental options that are not actually taken in vivo under normal conditions, and these priming signatures can be erased or reset during nuclear reprogramming. This makes it hard to support a strict one-to-one link between poised/primed states and fixed lineage fates [14,174]. Even for stringently defined pluripotency-associated poised enhancers, some fraction becomes active in the anticipated embryonic tissues, whereas others remain Polycomb-repressed, are preferentially deployed in distinct lineages, or fail to acquire H3K27ac altogether [30,55].

A plausible unifying view is that poised and primed marks encode a reservoir of developmental competence, a menu of accessible trajectories, rather than a hard-wired, deterministic lineage code. Within this broader interpretation, an important unresolved question is which contextual features, such as local sequence motifs, transcription factor availability, chromatin neighborhood or recent signaling inputs, bias specific pre-marked enhancers toward activation while others remain unused or become decommissioned. Addressing this problem will likely require prospective, single-cell, multi-omic and lineage-tracing strategies that follow individual poised elements across fate bifurcations, together with quantitative models that treat chromatin marks as one dimension within a higher-dimensional competence space rather than as standalone determinants of lineage choice.

### 7.2. Shadow and Multi-Enhancer Architectures Beyond Simple Redundancy

Drawing on the cases discussed above, we have emphasized that shadow enhancers can provide a major source of robustness by buffering random fluctuations and environmental perturbations, preserving expression thresholds, and stabilizing lineage decisions. As locus-specific dissection studies accumulate, however, it has become clear that what is often labelled “redundancy” is sometimes conditional or hierarchical, and often more complex than a simple backup model would imply (Figure 4B). Classic *Drosophila* work at the *snail* and *svb* loci exemplifies this point: as discussed above, deletion of either the primary or the shadow enhancer has little discernible effect under standard conditions, yet under thermal or dosage stress the same perturbations lead to impaired patterning and a failure to maintain normal phenotypes. This suggests that apparent redundancy under benign conditions may break down under excessive environmental stress [113]. Large-scale studies of shadow enhancer pairs show that many of these elements are under strong purifying selection, which is hard to square with the idea that they are simply interchangeable backups and instead suggests more distinct, context-limited roles [111]. At the *Sox9* testis locus, deletion of a single distal enhancer (Enh13) is sufficient to cause sex reversal despite the presence of additional testis enhancers, exemplifying a non-redundant, gatekeeper-like enhancer rather than one unit in an additive pool [175]. Similarly, sequential mutagenesis of the HoxD regulatory landscape reveals a functional stratification within enhancer clusters: some elements provide quantitative buffering of expression, others operate within narrow temporal or spatial windows, and still others execute unique, non-compensable functions. These patterns therefore argue for a hierarchical, division-of-labor organization rather than a pool of interchangeable enhancers [176].

Even among canonical shadow pairs, interactions are frequently non-additive. At *gap* gene loci, primary and shadow enhancers can antagonize each other to sharpen domain boundaries, such that loss of one module broadens expression rather than simply lowering dose [177]. Likewise, at the *snail* locus two *cis*-regulatory modules that are simultaneously active in vivo constrain each other’s spatial range and amplitude, so that deletion of one module can paradoxically increase expression in specific regions [178]. Taken together, these findings more simply indicate that multi-enhancer systems do not always operate as interchangeable backups and instead adjust transcription in context-dependent ways, including buffering, fine-tuning and shaping spatial or temporal expression patterns. Accordingly, while our review highlights multi-enhancer architectures, especially shadow pairs, as a key source of robustness, how general this buffering is and how strong it can be are still not fully clear. Improving the theory will require models that incorporate conditional redundancy, enhancer hierarchies, and non-linear interactions. These models should be guided by systematic perturbations across multiple regulatory regions and also by quantitative imaging across a wider range of loci and stress regimes.

### 7.3. Enhancer-Promoter Proximity: Instructive Driver or Permissive Scaffold?

In this review, we have discussed Polycomb-organized poised hubs and pre-configured E-P neighborhoods as “wired-for-timing” topologies that can accelerate gene activation once repression is relieved, particularly at neural developmental loci. Although pronounced lineage- and tissue-specific rewiring of E-P contacts have been documented at selected loci [81,179], many chromatin loops appear remarkably stable across tissues and are only weakly predictive of tissue-specific transcription, indicating that physical proximity alone is insufficient to specify transcriptional state (Figure 4C). Work in macrophage development illustrates this tension: a large fraction of E-P loops is pre-formed and maintained from progenitors to differentiated cells, whereas changes in H3K27ac and transcription factor binding on this relatively static scaffold, rather than wholesale rewiring of contacts, correlate with gene induction [180]. A similar picture emerges at the *Shh* limb locus, where the zone of polarizing activity regulatory sequence (ZRS) enhancer forms a robust pre-loop that persists before and after transcriptional onset; perturbing CCCTC-binding factor (CTCF) sites reduces contact frequency and lowers *Shh* expression, yet strong morphological defects appear only when this topological weakening is combined with a hypomorphic enhancer allele, such as subtle transcription factor motif optimizing variants within the ZRS [173,181]. This is consistent with 3D architecture acting as a permissive upper-bound modulator rather than a strictly instructive switch. Within this framework, switch-like instructive effects on developmental outcomes are more likely to arise when additional local alterations, such as shifts in transcription factor binding affinity or dosage accompany changes in 3D topology.

Other loci go further in decoupling activity from stable proximity. At the *Sox2* locus, live-cell imaging reveals enhancer-dependent transcriptional bursts that are not temporally aligned with episodes of E-P co-localization, arguing that sustained close contact is not required for enhancer function in that context [47]. Super-resolution studies at *Shh* during neural differentiation similarly show that enhancer activation can coincide with increased average distances between enhancers and promoters [182]. Developmental Capture-C in *Drosophila* further suggests that whether E-P topology behaves in a more permissive or a more instructive manner can itself vary across developmental stages and lineages, suggesting a shift from broadly similar, weakly predictive topologies during early cell-fate specification to more selective, expression-aligned contacts during later tissue differentiation [183]. These observations together provide a clearer and more intuitive view that proximity often creates a supportive environment for enhancer action but does not by itself dictate transcriptional outcome, and that the functional meaning of proximity depends strongly on developmental timing and cellular context.

In sum, a useful way to frame E–P contact hubs is as architectural “opportunity structures” that constrain where interactions can occur and how strongly loci respond, without necessarily dictating transcriptional state. A central question that remains is when proximity acts merely as a permissive scaffold, when it becomes rate-limiting or threshold-setting, and how these modes interface with enhancer strength, chromatin features, and the nuclear environment. Addressing this problem will likely require acute, locus-specific manipulation of 3D contacts combined with single-cell, time-resolved transcriptional readouts, enabling causal tests of sufficiency and necessity of proximity rather than continued reliance on correlation alone.

## 8. Conclusions

Across the developmental systems considered in this review, enhancer function can be parsimoniously framed along two intersecting axes: enhancer state dynamics, encompassing transitions among poised, primed, active, and latent configurations, and cooperative enhancer architecture, including modular and shadow arrangements. Viewed together, these axes motivate an “integrated enhancer logic” in which initial chromatin and transcription factor configurations predefine which regulatory options are even available, while ensembles of enhancers, embedded within three-dimensional chromatin neighborhoods, fine-tune the timing, amplitude, and robustness of gene activation during lineage decisions. If this logic can be systematically tested and formalized through the combined use of single-cell multi-omics, targeted perturbation, and quantitative modeling, current descriptive maps of chromatin and transcription could be elevated into predictive models of developmental trajectories, directly linking specific enhancer states and architectures to fate choice probabilities, transcriptional thresholds, and noise properties. In turn, such a predictive framework would provide a principled basis for interpreting how noncoding variants influence discrete state transitions or the stability of cooperative modules, and for designing temporally and cell-state-resolved perturbation strategies in enhanceropathies and other developmental disorders.

From this integrated perspective, a set of specific, experimentally testable expectations and challenges emerges. Poised and primed enhancers are unlikely to function as rigid, one-to-one codes for particular lineages; instead, they are best viewed as probabilistic reservoirs of competence, whose eventual deployment is conditioned by the surrounding transcription factor network, signaling environment, and three-dimensional nuclear context. Latent enhancers, by contrast, are expected to act primarily as stimulus-gated modules layered onto pre-existing lineage circuits, adjusting effector programs and trained responses rather than repeatedly resetting core identity. Cooperative multi-enhancer and shadow architectures may appear roughly additive under baseline conditions, yet under genetic or environmental stress they are predicted to exhibit nonlinear, strongly context-dependent behavior, thereby unmasking otherwise cryptic requirements for individual modules in maintaining thresholds, damping transcriptional noise, and sharpening expression patterns. In parallel, pre-formed E-P proximity is likely to operate in many settings as a permissive scaffold that delimits the ceiling and kinetics of transcriptional responses, with realized output determined by the combined state of enhancer chromatin, transcription factor occupancy, and cofactor recruitment dynamics imposed on this spatial framework. A central task going forward is therefore to delineate which combinations of sequence grammar, network architecture, nuclear milieu, and contact topology distinguish poised or primed elements that are productively engaged from those that remain latent or are actively decommissioned. A closely aligned goal is to move beyond static H3K4me1^+^ (±H3K27me3) catalogs toward quantitative competence maps that predict enhancer usage probabilities and branch choices along differentiation trajectories. In parallel, a broader accounting is needed of how enhancer modules combine across loci, tissues, developmental stages, and stress regimes, including when they behave additively, when their contributions are organized hierarchically, and when they interact antagonistically. Finally, it will be essential to resolve when changes in enhancer–promoter proximity represent genuinely rate-limiting, instructive events in fate specification versus permissive scaffolds or downstream consequences of shifts in enhancer state and transcription factor occupancy. Addressing these questions will require quantitative models that explicitly accommodate probabilistic enhancer usage, context dependence, and regulatory redundancy, together with high-resolution genomics, imaging, and perturbation. The framework synthesized in this review provides a conceptual scaffold for designing and interpreting such experiments, while also aiming to advance developmental gene regulation and enhancer-based disease toward more predictive and mechanistically grounded models.

## Figures and Tables

**Table 1:** Chromatin and transcriptional features of poised, primed, and active enhancers.

Type of Enhancers	Histone Mark and Variants	Enhancer RNA Expression	Target Expression
Poised enhancer	H3K27me3	Extremely low	Extremely low
	H3K4me1		
	H2A119ub1		
	H2A.Z		
Primed enhancer	H3K4me1	Low	Low
Active enhancer	H3K27ac	High	High
	H3K14ac		
	H3K9ac		

## Data Availability

No new data were created or analyzed in this study.

## Abbreviations

The following abbreviations are used in this manuscript:

TF	Transcription factor
Pol II	RNA polymerase II
H3K4me1	Histone H3 lysine 4 mono-methylation
H3K27ac	Histone H3 lysine 27 acetylation
H3K9ac	Histone H3 lysine 9 acetylation
H3K14ac	Histone H3 lysine 14 acetylation
CBP	CREB-binding protein
p300	EP300
eRNA	Enhancer RNA
enhancer-promoter	E-P
BRD4	Bromodomain-containing protein 4
H3K27me3	Histone H3 lysine 27 tri-methylation
H2AK119ub1	Histone H2A lysine 119 ubiquitylation
H2A.Z	H2A histone family, member Z
FAIRE-seq	Formaldehyde-assisted isolation of regulatory elements sequencing
ATAC-seq	Assay for transposase-accessible chromatin using sequencing
ChIP-seq	Chromatin immunoprecipitation sequencing
CUT&RUN	Cleavage under targets and release using nuclease
CUT&Tag	Cleavage under targets and tagmentation
3C	Chromosome conformation capture
4C	Circular chromosome conformation capture
Hi-C	High-throughput chromosome conformation capture
Micro-C	Micrococcal nuclease–based chromosome conformation capture
TAD	Topologically associating domains
MPRA	Massively parallel reporter assays
STARR-seq	Self-transcribing active regulatory region sequencing
CRISPR	Clustered regularly interspaced short palindromic repeats
CRISPRi	CRISPR interference
CRISPRa	CRISPR activation
LoF	loss-of-function
ERα	estrogen receptor alpha
HDAC	Histone deacetylase
H2BNTac	H2B N-terminal acetylation
Notch	Neurogenic locus notch homolog protein
AP-1	Activating protein-1
BAF/SWI-SNF	Switch/Sucrose non-fermentable
BRG1	Brahma-related gene 1
PRC1	Polycom repressive complex 1
PRC2	Polycom repressive complex 2
KDM6A/UTX	Lysine-specific demethylase 6A
KDM6B/JMJD3	Lysine-specific demethylase 6B
MLL4/KMT2D	Histone lysine N-methyltransferase 2D
GATA4	GATA binding protein 4
NKX2-5	NK2 homeobox 5
SRF	Serum response factor
TBX5	T-box transcription factor 5
CGI	CpG island
Hox	Homeobox
PcG	Polycomb group
mESC	Mouse embryonic stem cell
LDTF	Lineage-determining transcription factor
FOXA	Forkhead box protein A
PDX1	Pancreatic and duodenal homeobox 1
LSD1 (KDM1A)	Lysine-specific demethylase 1
LPS	Lipopolysaccharide
IFN-γ	Interferon-γ
IL-4	Interleukin-4
NF-κB	Nuclear factor kappa-light-chain-enhancer of activated B cells
MLL3 (KMT2C)	Histone lysine N-methyltransferase 2C
TNFα	Tumor necrosis factor alpha
LCR	Locus control region
GATA2	GATA binding protein 2
Fgf8	Fibroblast growth factor 8
AER	Apical ectodermal ridge
Bcl11b	B-cell lymphoma 11B
Runx1	Runt-related transcription factor 1
Irf8	Interferon regulatory factor 8
Sox10	SRY-box transcription factor 10
fos	FBJ osteosarcoma oncogene
Ets1	ETS proto-oncogene 1
Pax6	Paired box 6
MEIS	Myeloid ecotropic viral integration site
Shh	Sonic hedgehog
MRCS1	Mammal reptile conserved sequence 1
MFCS4	Mammal fish conserved sequence 4
ATOH7	Atonal BHLH transcription factor 7
SOX2	SRY-box transcription factor 2
POU5F1	POU domain, class 5, transcription factor 1
MEF2	Myocyte enhancer factor 2
MYH7	Muscle myosin 7
TTN	Titin
SCN5A	Sodium voltage-gated channel alpha subunit 5
COUP-TFII	COUP transcription factor 2
Scn10a	Sodium voltage-gated channel alpha subunit 10
Cx30.2	Connexin 30.2
Hcn4	Hyperpolarization-activated cyclic nucleotide gated potassium channel 4
TBX3	T-box transcription factor 3
WT1	WT1 transcription factor
ISL1	Insulin Gene Enhancer Protein ISL-1
TBX18	T-box transcription factor 18
PU.1	spi-1 proto-oncogene
C/EBP	CCAAT/enhancer-binding protein
E2A	E2A transcription factor
IRF	Interferon regulatory factor
HSC	Hematopoietic stem cell
NEUROD1	Neurogenic differentiation 1
ASCL1	Achaete-scute family bHLH transcription factor 1
Sox9	SRY-box transcription factor 9
ZRS	Zone of polarizing activity regulatory sequence
CTCF	CCCTC-binding factor
